# Correction: Sexual dichromatism in the neotropical genus *Mannophryne* (Anura: Aromobatidae)

**DOI:** 10.1371/journal.pone.0238962

**Published:** 2020-09-03

**Authors:** Mark S. Greener, Emily Hutton, Christopher J. Pollock, Annabeth Wilson, Chun Yin Lam, Mohsen Nokhbatolfoghahai, Michael J. Jowers, J. Roger Downie

There are errors in affiliation 4 for author Michael J. Jowers. The correct affiliation 4 is: CIBIO/InBIO (Centro de Investigação em Biodiversidade e Recursos Genéticos), Universidade do Porto, Vairão, Portugal.

## References

[1] Greener MS, Hutton E, Pollock CJ, Wilson A, Lam CY, Nokhbatolfoghahai M, et al (2020) Sexual dichromatism in the neotropical genus Mannophryne (Anura: Aromobatidae). PLoS ONE 15(7): e0223080 10.1371/journal.pone.0223080 32639962 PMC7343140

