# Supplementary material for: Sexual dichromatism in the neotropical genus Mannophryne (Anura: Aromobatidae)
Source: PLoS One. 2020 Jul 8;15(7):e0223080. doi: 10.1371/journal.pone.0223080 (PMC7343140; doi:10.1371/journal.pone.0223080)
Supplement: S1 Table — Data for the number of individuals sampled from each site per year. (DOCX) [file pone.0223080.s001.docx]

**S1 Table. Frog collection data**.

| **Site** | **Lat** | **Long** | **Sample Size** | | |
| --- | --- | --- | --- | --- | --- |
|  |  |  | **2015** | **2016** | **2018** |
| Asa Wright Nature Centre | 10.71989 | -61.2975 |  |  | 13 |
| Brasso Seco | 10.73873 | -61.2852 | 11 | 35 |  |
| Caura 1 | 10.66758 | -61.3683 |  |  | 11 |
| Caura 2 | 10.67418 | -61.3692 | 18 | 165 |  |
| Edith Falls | 10.72105 | -61.6277 | 14 | 35 |  |
| Las Cuevas | 10.76727 | -61.4147 | 20 | 33 |  |
| Lopinot Valley | 10.68233 | -61.3277 |  |  | 13 |
| Maracas Bay | 10.75939 | -61.4245 | 19 | 45 | 23 |
| Maracas Waterfall | 10.72958 | -61.403 | 23 | 34 |  |
| Mount St Benedict | 10.66827 | -61.3998 | 18 | 31 | 21 |

Only data from frogs collected in 2015 and 2016 were included in throat colour and collar variability analysis. Frogs collected in 2018 were used in the escape response experiment.
