# Supplementary material for: Sexual dichromatism in the neotropical genus Mannophryne (Anura: Aromobatidae)
Source: PLoS One. 2020 Jul 8;15(7):e0223080. doi: 10.1371/journal.pone.0223080 (PMC7343140; doi:10.1371/journal.pone.0223080)
Supplement: S2 Table — Breakdown of the significance values from each statistical test conducted. (DOCX) [file pone.0223080.s002.docx]

**S2 Table. Significance values from analysis of escape response trials.**

|  | F | *p* |
| --- | --- | --- |
| Site ~ R:G Ratio | 2.4714 | 0.05155 |
| R:G Ratio ~ Weight | 18.416 | 4.992e-05 |
| R:G Ratio ~ SVL | 19.071 | 3.782e-05 |
| Weight ~ SVL | 969.91 | 2.2e-16 |
| **Site and Escape response** | | |
| Site ~ Maximum Distance | 0.6505 | 0.629148 |
| **Temperature and Escape response** | | |
| Temperature ~ Maximum Distance | 0.8911 | 0.349635 |
| **Humidity and Escape Response** | | |
| Humidity ~ Maximum Distance | 0.0016 | 0.968629 |
| **Throat colour and Escape response** | | |
| R:G Ratio ~ Maximum Distance | 11.5539 | 0.001217 |
| R:G Ratio ~ Total Distance | 2.7417 | 0.1033 |
| R:G Ratio ~ Minimum Distance | 0.6617 | 0.41974 |
| R:G Ratio ~ Mean Distance | 3.0922 | 0.08433 |
| R:G Ratio ~ Initial Jump Distance | 3.9728 | 0.05103 |
