# Supplementary material for: Sexual dichromatism in the neotropical genus Mannophryne (Anura: Aromobatidae)
Source: PLoS One. 2020 Jul 8;15(7):e0223080. doi: 10.1371/journal.pone.0223080 (PMC7343140; doi:10.1371/journal.pone.0223080)
Supplement: S4 Table — All data collected and used for analysis in colour development in juvenile Mannophryne trinitatis study. (DOCX) [file pone.0223080.s004.docx]

**Ontogeny of sexual dichromatism in *M. trinitatis***

1. **Females** (**determined by development of yellow throat colour**)

Days followed(post-meta) Final SVL(mm) First time (days) yellow seen SVL at first yellow

127 17.2 38 15.3

119 17.8 30 15.6

116 16.8 61 17.4

112 16.7 57 16.9

109 18.0 54 16.3

106 17.0 54 16.3

105 17.0 50 16.3

105 16.2 50 15.1

103 15.4 76 15.7

102 17.4 47 15.4

102 16.6 47 16.0

98 15.8 43 15.6

97 15.2 42 15.2

96 14.9 41 13.8

96 16.8 69 15.0

131 14.6 61 13.1

131 15.8 61 15.3

129 15.1 59 15.5

Mean 110+/- 16.4+/- 52.2+/- 15.5+/-

+/- SD 12.0 1.0 11.1 1.0

(n= 18. NB two females excluded because they were not followed beyond 90 days; but included in sex ratio calculation)

1. **Males (no change to grey throat, dark collar)**

143 18.7

133 18.0

119 17.1

138 16.2

129 16.4

(n=5) 132+/-8.2 17.3+/-0.9
